# HACCP-Based Programs for Preventing Disease and Injury from Premise Plumbing: A Building Consensus

**DOI:** 10.3390/pathogens4030513

**Published:** 2015-07-09

**Authors:** William F. McCoy, Aaron A. Rosenblatt

**Affiliations:** 1 Phigenics, LLC, 1701 Quincy Ave., Suite 32, Naperville, IL 60540, USA; 2 Gordon & Rosenblatt, LLC, 45 Rockefeller Plaza, 20th Floor, New York, NY 10111, USA; E-Mail: ar@gordonrosenblatt.com

**Keywords:** *Legionella*, Legionnaires’ disease, legionellosis, non-tuberculous mycobacteria (NTM), *Pseudomonas*, biofilm, free living amoeba (FLA), free living protozoa (FLP), HACCP, buildings, water safety plans, hazards

## Abstract

Thousands of preventable injuries and deaths are annually caused by microbial, chemical and physical hazards from building water systems. Water is processed in buildings before use; this can degrade the quality of the water. Processing steps undertaken on-site in buildings often include conditioning, filtering, storing, heating, cooling, pressure regulation and distribution through fixtures that restrict flow and temperature. Therefore, prevention of disease and injury requires process management. A process management framework for buildings is the hazard analysis and critical control point (HACCP) adaptation of failure mode effects analysis (FMEA). It has been proven effective for building water system management. Validation is proof that hazards have been controlled under operating conditions and may include many kinds of evidence including cultures of building water samples to detect and enumerate potentially pathogenic microorganisms. However, results from culture tests are often inappropriately used because the accuracy and precision are not sufficient to support specifications for control limit or action triggers. A reliable negative screen is based on genus-level Polymerase Chain Reaction (PCR) for *Legionella* in building water systems; however, building water samples with positive results from this test require further analysis by culture methods.

## 1. Introduction

Every year, tens of thousands of preventable injuries and deaths are caused by exposure to microbial, chemical and physical hazards from building water systems [1,2,3,4,5,6,7,8]. Inhalation by susceptible persons of pathogen-rich bio-aerosols from premise plumbing can result in life-threatening, sometimes fatal infections. The control of microbial hazards associated with building water systems is particularly challenging, made all the more difficult by practical limits on hot water temperatures owing to scalding risks.

Plumbing-associated microorganisms of concern include bacteria, fungi and protozoa. The bacteria *Acinetobacter*, *Elizabethkingia (Flavobacterium)*, *Escherichia coli*, *Klebsiella*, *Legionella*, non-tuberculous Mycobacteria (NTM), *Pseudomonas*, and *Stenotrophomonas* are known to cause significant disease associated with building water systems. Pathogenic fungi associated with plumbing include *Aspergillus* and *Fusarium.* The protozoans *Acanthamoeba* and *Vermamoeba* (renamed from *Hartmanella vermiformis*) can themselves cause disease; they also are hosts for parasitic pathogenic bacteria in building water systems and play a defining role in bacterial virulence, proliferation and transmission [9,10,11,12,13,14,15,16,17,18,19,20,21,22,23,24].

The only plumbing-associated disease requiring notification in the US is legionellosis [8], a severe pneumonia caused by the bacterium *Legionella.* Building water systems are now recognized as the primary source of legionellosis [1,2,7]. The US Centers for Disease Control and Prevention (CDC) has estimated there are as many as 18,000 cases of Legionnaires’ disease annually; the US Occupational Safety and Health Administration (OSHA) has estimated that Legionnaires’ disease results in about 4000 deaths in the US each year [6,8]. Other plumbing-associated pathogens, such as *Pseudomonas*, may cause as much or more disease as *Legionella,* but lack of required reporting and other factors make quantification difficult [1].

Potable water supplied through community water systems in the US is treated to *National Primary Drinking Water Standards*, a set of requirements developed by the United States Environmental Protection Agency (USEPA) under authority of the *Safe Drinking Water Act* (SDWA). Generally, SDWA-compliant water is safe for its intended use. However, it is not sterile; it contains small numbers of microorganisms, which makes it unsuitable for certain uses. For example, tap water should not be used for purposes that require sterile solution, such as for sinus irrigation or soaking contact lenses. Microorganisms in high-quality, SDWA-compliant drinking water can enter plumbing systems in small numbers, attach to the inside surfaces of pipes and equipment, form biofilms and amplify to very large, potentially-dangerous numbers [12,25]. The complex microbial ecosystems that characterize natural biofilms and the inter-relationships of biofilm-related organisms are fundamental factors that underlie disease from plumbing-associated pathogens [1,7,12,25].

Building water systems are especially susceptible to biofilm development and microbial colonization. Premise plumbing in buildings typically is an extensive network of small diameter pipes with high surface-to-volume ratios. Flow is often sporadic; water can stagnate and become tepid with levels of residual disinfectant dropping off quickly to dangerously low levels. Nutrients required by microorganisms are available from plumbing materials, sediment and additives, such as phosphate corrosion inhibitors. Taken together, these conditions facilitate incubation of microorganisms and extensive, sometimes-rapid colonization of surfaces. Pathogenic biofilms established on interior surfaces of plumbing infrastructure may shed or be dislodged, then be broadcast as respirable droplets in infectious bio-aerosol from the plumbing into the environment, for example through showerheads, faucet fixtures and ornamental fountains. Similarly, they may be released from non-potable water in cooling towers in the HVAC system of the building [1,7,12,25].

The physical-chemical parameters that are conducive to biofilm development and microbial amplification—temperature, pH, water age—are reasonably well known for prominent plumbing-associated pathogens, such as *Legionella*, as are the parameters that inhibit or prevent such amplification [19].

### 1.1. Building Water Systems are Comprised of a Series of Unit Processes

Water in buildings is processed in many ways. For example, it may be conditioned, filtered, stored, heated, cooled, pressure regulated and distributed through fixtures that automatically restrict flow and temperature. Often, processing degrades the quality of the water, increasing the likelihood of biofilm formation and microbial amplification. Building water systems are comprised of a series of processes that operate within definable parameters that can be measured and managed in real time. Therefore, prevention of disease and injury can be accomplished by maintaining the physical-chemical environment of each process in the plumbing system at conditions known to inhibit or prevent microbial amplification.

Process management programs are widely applied to control environmental-source disease across many fields including food, pharmaceuticals and bottled-water production. Formalized process management approaches used to ensure product safety trace back to production process monitoring methods, such as Failure Modes and Effects Analysis (FMEA) developed during WWII in connection with the manufacture of artillery shells, where end-of-pipe testing was not practical. [26]. FMEA gave rise to a number of related methodologies, including Hazard and Operability Studies (HAZOP), Hazard Vulnerability Analysis (HVA) and Hazard Analysis and Critical Control Point (HACCP).

### 1.2. History of Hazard Analysis and Critical Control Point

In the early 1960s the Pillsbury Company, working in close collaboration with the National Aeronautic and Space Administration (NASA) and the US Army Laboratories, developed a formal product safety methodology based on process management. The initial purpose was to develop a system for assuring the safety of food and water provisions to be sent into space with manned space missions. Shortly thereafter, Pillsbury adopted HACCP for its own production of commercial food products.

In 1969, under contract to FDA, Pillsbury developed a training program called “Food Safety through the Hazard Analysis and Critical Control Point System”, which appears to be the earliest published use of the term “HACCP”. The HACCP system, which initially comprised three principles, continued to evolve. In 1987, the National Advisory Committee on Microbial Criteria for Foods (NACFM) was formed and given responsibility for working with other organizations, such as the Codex Committee for Food Hygiene, to harmonize various HACCP initiatives. By 1997—notwithstanding slight differences in terminology used by NACFM, Codex, FDA and others—general consensus was reached on the seven principles that define HACCP [27].

HACCP provides a science-based approach to ensuring product safety, by the systematic identification of specific hazards and the implementation of measures for their control. Control measures must be described in detail, and all details of implementation must be recorded in order (a) to document that the controls are being applied as intended and (b) that, when applied as intended, controls are effective in controlling the identified hazards. HACCP is compatible with the ISO 9000 series and other quality management systems. The seven principles of HACCP [27] are:

(1) Conduct a Hazard Analysis;
(2) Determine the Critical Control Points;
(3) Establish Critical Limit(s);
(4) Establish a system to monitor control of the Critical Control Points;
(5) Establish Corrective Action(s) to be taken when monitoring indicates that a particular Critical Control Point is not within Critical Limits;
(6) Establish procedures to confirm that the HACCP system is working effectively; and
(7) Establish documentation of all procedures pertaining to these HACCP principles and their application.

There are twelve steps for implementing the seven principles of HACCP:

(1) Establish a HACCP team;
(2) Describe the system;
(3) Identify intended use(s);
(4) Construct process flow diagrams;
(5) Confirm the accuracy of the process flow diagrams;
(6) List all potential hazards associated with each process step, conduct a hazard analysis and consider measures to control identified hazards at each step;
(7) Determine Critical Control Points, the locations where control must be applied to prevent hazards from causing harm;
(8) Establish Critical Limits for each Critical Control Point;
(9) Establish monitoring procedures for each Critical Control Point and specify frequency of monitoring;
(10) Establish corrective actions to be taken when monitoring indicates that conditions at a Critical Control Point are outside of Critical Limits;
(11) Establish procedures to confirm that the plan is being implemented as designed (verification) and is working effectively (validation); and
(12) Establish documentation and record keeping procedures.

Application of these seven HACCP principles and twelve steps has become the standard best practice for food safety management systems around the world [27] and is mandated in the US for certain food production processes, though most food safety applications of HACCP in the US are voluntary [7].

## 2. HACCP for Protection of the Public Water Supply

The beneficial use of HACCP is not limited to any specific type of hazard or process; the methodology has proved effective and flexible, and is increasingly applied to industries other than food, including cosmetics, pharmaceuticals and bottled water.

The application of HACCP to public water supplies was first proposed in 1994 by researchers affiliated with the World Health Organization [28]. In a number of initiatives that followed, HACCP principles were applied to the broader control of infectious disease from water, and provided the basis for the Water Safety Plan (WSP) approach in the third edition of the WHO *Guidelines for Drinking-water Quality* [3]. In describing the WSP paradigm, WHO used terminology that differed somewhat from that used in the standardized HACCP literature, which dealt primarily with food. However, the close correspondence between the WSP construct and HACCP has been observed by a many authors and in fact, the WHO *Guidelines for Drinking-Water Quality* has been described as “a way of adapting the HACCP approach to drinking water systems” [29]. Several countries have adopted HACCP-based guidelines. For example, in Denmark, the Danish Water and Waste Water Association (DWWA) developed guidelines for water safety based on the WSP and HACCP principles. The DWWA guidelines include the complete drinking water system from source to tap, including private installations. There are many published examples of successful applications [30,31,32,33,34,35,36].

## 3. HACCP-Based Programs for Preventing Disease and Injury from Building Water Systems

Scientists from the US Centers for Disease Control and Prevention (CDC) have investigated hundreds of Legionnaires’ disease outbreaks since 1977. They have observed that practices at the majority of facilities that experienced outbreaks, though diverse, had several striking similarities:

- They lacked documentation of building water systems and familiarity with water processes, especially in large, complex systems;
- They lacked a systematic program for identifying, monitoring and controlling factors known to affect microbial growth (e.g., water temperatures, disinfectant residual levels); and
- They lacked inter-disciplinary/inter-departmental communication, e.g., between facility managers and clinicians.

Since 2000, the CDC Legionnaires’ disease Outbreak Response Team has recommended HACCP-based practices for facilities that have been associated with outbreaks of Legionnaires’ disease. There have been no subsequent outbreaks in buildings following the recommended protocols (personal communication, C. Lucas, CDC, 2012).

In 2010, a HACCP water management program was developed and implemented at the Mayo Clinic in Rochester, MN yielding significant improvements in building water system safety [37].

In 2004, WHO published “*Guidelines for Drinking-water Quality*”, in which HACCP principles were organized into a formalized, water-oriented construct called the Water Safety Plan (WSP) [3]. In 2007, WHO published “*Legionella and the Prevention of Legionellosis*”, in which the WSP framework was adapted to address a single hazard, *Legionella* in building water systems [4]. In its recommendations for control of *Legionella* and prevention of legionellosis, WHO determined that *Legionella* testing is not a suitable control measure for several reasons, including un-reliability of culture methods, inherent time delays that render sampling/culture useless for operational monitoring and differences between culture requirements for different *Legionella* species. Rather, WHO recommends that prevention of legionellosis should be accomplished by operational monitoring and control measures that provide real-time results (e.g., monitoring of biocide concentrations, temperature and pH).

In 2011, WHO extended its adaptation of HACCP in WHO published “*Water Safety in Buildings*”, expanding the scope of the WSP paradigm to include multiple hazards associated with premise plumbing [5].

In 2013, NSF International, a leading public health and safety NGO, initiated an education and training program, “*HACCP for Building Water Systems*”. The course built on the foundation established by WHO, embracing an all-hazards approach to prevention of disease and injury associated with building water systems. In contrast to the WSP construct, the NSF course embraced traditional HACCP terminology [38].

There is continuing debate on the advantages of using traditional HACCP terminology *vs.* new, special-purpose coinages for HACCP-based methodologies that address building water systems. However, there is a remarkable consensus that programs incorporating established HACCP principles and steps can effectively prevent disease and injury from plumbing-associated pathogens and other hazards. This consensus developed based on widespread recognition of certain features of HACCP-based programs:

- HACCP-based methodology enjoys the benefit of extensive real-world use in mitigating a range of environmental risks, including microbial hazards associated with public water supplies.
- HACCP-based methodology is well suited to establishing and maintaining appropriate controls of temperature, disinfectant residual and other factors that can reduce environmental exposure of building occupants, especially susceptible persons, to large numbers of plumbing-associated pathogens.
- HACCP-based methodology provides a systematic, standardized framework that can accommodate substantial variation in buildings and building water systems, including differences in purpose, design and propensity for disease transmission.
- HACCP-based methodology provides a systematic, standardized framework for risk characterization, hazard prevention and validation but does not prescribe specific means or methods. It is designed to accommodate future scientific progress and new/improved methods.
- HACCP-based methodology provides a practical, resource-efficient way for the largest number of buildings to accomplish significant risk reduction at reasonable cost. It enables technically competent building personnel to implement an effective hazard-prevention plan without reliance on expensive consultants and other specialists.
- HACCP-based programs for management of water systems in buildings have been developed and proposed by government agencies (VHA), major industry groups (ASHRAE) and prominent public health organizations (NSF International). They share fundamental features of HACCP methodology, with small differences in terminology and level of prescription. Except where noted (Table 1), these programs use conventional HACCP terminology.

**Table 1:** Comparison of HACCP-based programs for building water system management.

Program Components	NSF Int’l * 444	WHO * WSP	VHA * Directive 1061	ASHRAE * 188
Interdisciplinary Team with authority & responsibility	+	+	+	+
Water system description (process flow diagrams)	+	+	+	+
Hazard analysis and risk characterization based on water system description	+	+ *Note*: Variously called hazard analysis or risk assessment	+ *Note*: Risk characterization includes assessment of clinical and environmental factors	+
Critical Control Points are selected based on hazard analysis and risk characterization	+	+	+ *Note*: Controls are called “Engineering Controls”. Values are prescribed for temperature and oxidant residual levels	+ *Note*: Critical Control Points are called “Control Locations”
Critical Limits are specified and monitored; Corrective actions are required	+	+	+	+ *Note*: Critical Limits are called “Control Limits”
Confirmation that the plan is being implemented according to design (verification) is required	+	+	+	+
Confirmation that controls, when applied according to plan, are effectively controlling hazards (validation) is required	+ *Note*: Both initial and ongoing validation are required	+ Note: Validation is variously called monitoring or testing	+ *Note*: Requires validation by both environmental and clinical testing. Responses to test results are prescribed	+

* Abbreviations: HACCP is “Hazard Analysis and Critical Control Point”; WSP is “Water Safety Plan” (WHO); VHA is Veterans Health Administration; ASHRAE = American Society of Heating, Refrigerating and Air-conditioning Engineers; NSF International.

### 3.1. WHO Water Safety Plans

**Figure 1:**
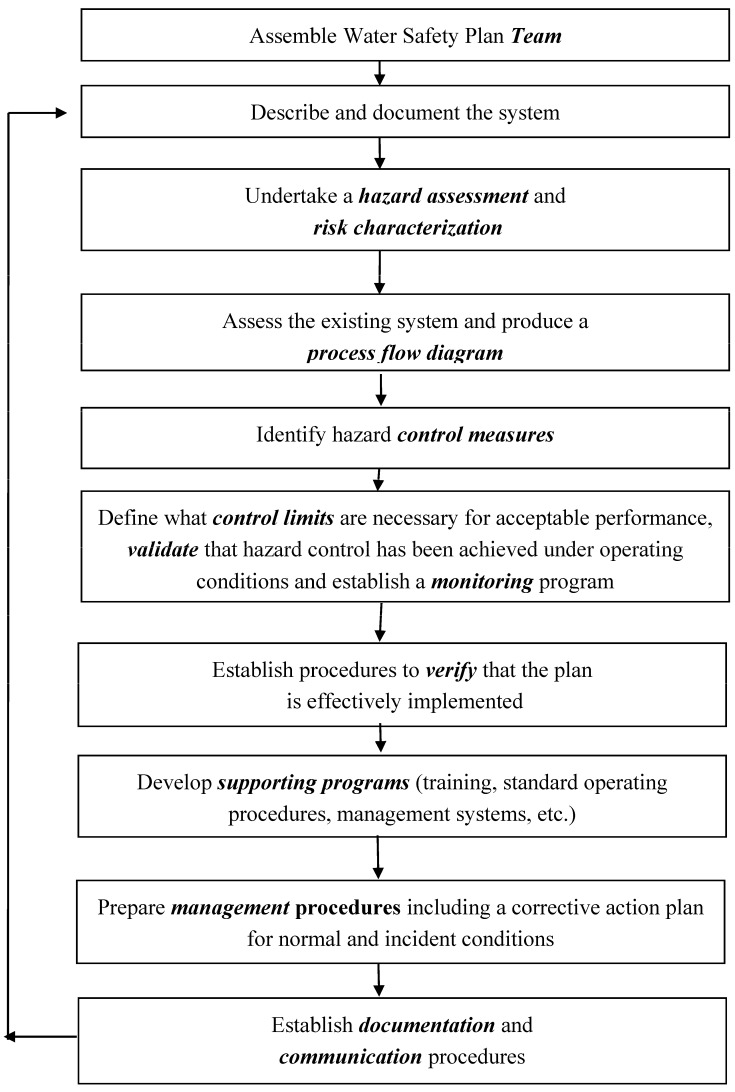
Steps in the development of a Water Safety Plan (WSP) as recommended by the World Health Organization. Text in bold indicate key concepts in the WSP hazard analysis and control system.

There is no fundamental difference between WHO-WSP paradigm and HACCP; however, the term “critical control point” is not used by WHO in connection with Water Safety Plans. Rather, WHO simply provides that control measures and the locations at which they are to be applied must be specified in the WSP. There are slight differences in language, for instance the term “hazard assessment” is used instead of the HACCP term “hazard analysis” (Figure 1).

### 3.2. VHA Directive 1061

In 2014, the US Veterans Health Administration (VHA) revised its Legionnaires’ disease prevention plan directive to align required best practice, including HACCP principles, in all of its healthcare facilities [39]. The VHA Directive addresses only Legionnaires’ disease and scalding. Going beyond providing a methodological framework, the VHA Directive (a) requires the Team consider clinical and environmental factors in developing risk characterizations, (b) requires testing of both clinical and environmental samples for validation, (c) prescribes specific responses based on test results, and (d) prescribes specific values for water temperature and residual oxidant. It calls control measures “engineering controls”.

### 3.3. ANSI/ASHRAE 188-2015

On 26 June 2015, the American Society of Heating, Refrigerating and Air-conditioning Engineers (ASHRAE) published Standard 188-2015: “*Legionellosis: Risk Management for Building Water Systems*”. The standard practice, ANSI/ASHRAE 188-2015, addresses disease and injury from only one pathogen: *Legionella*. All of the HACCP principles are fully utilized with a few HACCP terms adapted with alternative words. For example, the term “Control Locations” is used instead of “Critical Control Points”; “Control Limits” is used instead of “Critical Limits”; “Water Management Program” is used instead of “HACCP Plan”. A separate normative appendix for qualifying health care facilities is provided: it utilizes terminology consistent with that used in the health care regulatory environment but retains entirely all of the HACCP principles.

### 3.4. BSR/NSF 444

In February 2014, NSF International filed with the American National Standards Institute (ANSI) a project initiation notification (PIN) signaling its intent to make a consensus standard *BSR/NSF 444*, “*Prevention of Injury and Disease Associated with Building Water Systems*”. Draft Standard 444 will be an “all hazards” standard, addressing physical, chemical and microbial hazards associated with premise plumbing. As of this writing, NSF International is forming a committee to develop *BSR/NSF 444*. The details of 444 will be determined by the committee following ANSI procedures; the final form of the proposed standard cannot be known until the standard making process, including required public review, is complete. However, NSF has prepared a preliminary “starting” draft, which is substantially consistent with its education and training program, “*HACCP for Building Water Systems*”. The preliminary draft uses traditional HACCP terminology, and provides a rigorous but non-prescriptive methodological framework for hazard identification, analysis and control.

Table 1 shows a comparison of HACCP-based programs for building water system management.

## 4. Validation

Perhaps the most controversial and potentially confusing aspect of building water system management is validation: proof that hazards have been controlled under operating conditions. Validation can include many kinds of evidence. For example, disease surveillance data and measurements of physical and/or chemical parameters at sentinel points in the facility provide evidence of hazard control under operating conditions. Testing for waterborne pathogens in samples taken from building water systems can also be useful validation evidence. However, it must be noted that culture test methods for potentially pathogenic microorganisms in building water are fraught with analytical difficulties and must, therefore, be used with caution [40,41,42].

For instance, the limit in the *Legionella* spread plate test is about 10 CFU/mL; numerical values below this are not reliable. In the CDC laboratory proficiency program (ELITE), member laboratories are sent standardized samples to analyze and report back results. ELITE samples that contain less than 10 CFU/mL are scored as “variable” by the CDC reference lab because *Legionella* is not reliably detected at concentrations lower than that. The results were independent of whether the sample was pure or mixed, positive or variable and indicated that 10 CFU/mL is at or near the lower limit of detection [41,42].

The most common approach to improving the sensitivity of the spread plate method is by concentrating the water sample using vacuum filtration. A quantity of water is filtered; the filter is manipulated to remove all the material captured by the filter (the “filtrate”); the filtrate is then suspended in a volume of water smaller than the sample volume filtered. The ratio of these volumes therefore determines the concentration factor. For example, if 100 mL sample is filtered and then the filtrate is re-suspended in 10 mL of sterile water, then the sample has by this means been concentrated 10×. If 1000 mL is filtered and re-suspended into 10 mL of water, the concentration factor is 100×. Since the limit of detection without any concentration is 10 CFU/mL, the example given here would result in theoretical detection limits of 1 CFU/mL and 0.1 CFU/mL, respectively.

However, these manipulations of the sample to concentrate it result in significant changes. There are usually many kinds of microorganisms, including the free-living amoebic (FLA) host cells of *Legionella* in samples taken from building water systems. In a recent survey of utility non-potable water, every *Legionella*-positive sample was also positive for *Acanthamoeba* [43]. In many samples, particles and pieces of biomass are homogenized. Biomass from these samples is concentrated then re-suspended into a “puree”. This milieu is a dense and dynamic microcosm of living microorganisms in competition.

**Figure 2:**
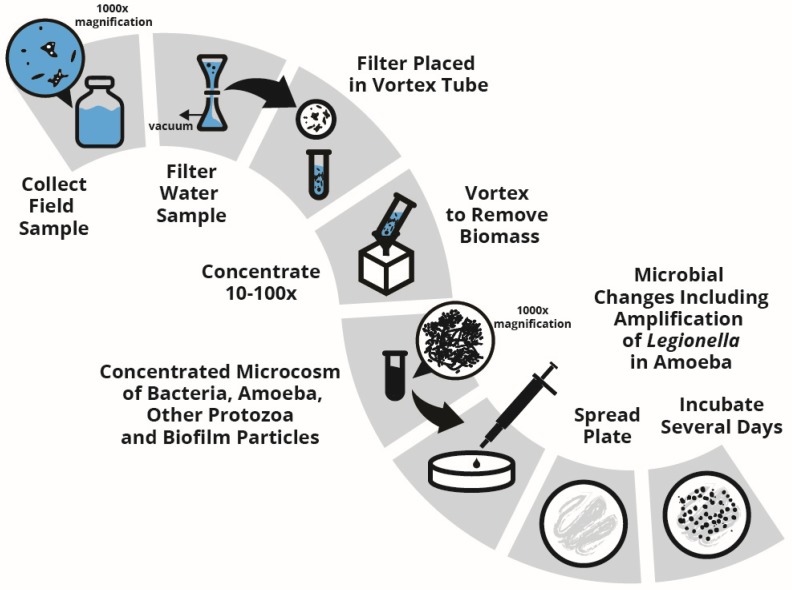
Schematic of spread plate method for detection and enumeration of *Legionella* and other potentially pathogenic bacteria from building water systems. Highly concentrated biomass from building water systems is a puree of bacteria, amoeba and other protozoa that, after incubation for several days in optimal conditions for growth, transform the spread-plated building water sample into a specimen with questionable resemblance to the sample when it was first obtained. Changes that occur on the plates during incubation include amplification of intracellular pathogens (such as *Legionella* and *Mycobacterium*) and consumption of potentially pathogenic bacteria by grazing protozoa on the plates. These changes render results difficult to interpret.

The rich concentrated milieu is then spread onto a nutrient-rich plate and incubated in optimal conditions for several days to promote growth (Figure 2). During this incubation period, *Legionella* and other parasitic bacteria may infect amoeba and grow to massively high numbers on plates [44]. Rapid growth of *Legionella* in their natural FLA hosts into detectable colonies on agar plates is well known [45]. The resultant high count of *Legionella* on a spread plate may have little or no resemblance to the concentration of *Legionella* in the water sample when it was removed from the building [42].

Therefore, accuracy and precision of results from spread plate cultures are not sufficient to support specifications for hazard control. The precision (standard deviation) of numerical results from spread plate analyses is poor: differences in numerical values less than about an order of magnitude are not statistically significant. Results from culturing building water systems should not be used to specify control levels or actions.

Despite these analytical difficulties, “detect/no-detect” (“positive or negative”) results from spread plate culture methods were reliable when compared to results from a reference laboratory at about or above 10 CFU/mL [41,42].

### Negative Screening of Environmental Samples with the PCR for *Legionella*

Since it has been established that the binary (“positive or negative”) interpretation of culture results above about 10 CFU/mL is scientifically defensible, therefore a practical and reliable negative screen to quickly identify culture-negative samples would useful. A reliable negative screen would significantly save both money and time. It would not, of course, eliminate the need for further analysis of positive samples.

The *Legionella* PCR negative screen can be recommended for building water samples. Genus-level Polymerase Chain Reaction (PCR) testing for *Legionella* in building water systems has been shown to be a practical and reliable negative screen. In a study of 3708 building water samples split for analyses by spread plate culture and PCR using materials and method previously published [42], the negative predictive value of PCR results was just under 97% (Table 2). Rapid screening of culture-negative samples is beneficial because it saves both time and analytical costs.

However, building water samples from which positive PCR detections are obtained require further analysis. A PCR positive result can be due to (1) viable, (2) viable but not culturable (VBNC), and/or (3) non-viable (killed) *Legionella* in the sample. Because of this, the Positive Predictive Value of PCR from analysis of 3708 building water samples was very poor (Table 2).

**Table 2:** Binary statistical analysis of Polymerase Chain Reaction (PCR) results for *Legionella* detection in samples shipped overnight from building water systems.

Binary Statistical Parameter	Total (Potable + Utility) Water Samples	Utility Water Samples	Potable Water Samples
True-Positives ^1^	520	38	482
False-Positives	765	167	598
True-Negatives	2342	365	1977
False-Negatives	81	9	72
Sum	3708	579	3129
Accuracy (%) ^2^	77.2	69.6	78.6
Specificity (%) ^3^	75.4	68.6	76.8
Sensitivity (%) ^4^	86.5	80.9	87.0
Positive Predictive Value (%) ^5^	40.5	18.5	44.6
Negative Predictive Value (%) ^6^	96.7	97.6	96.5

^1^ “True-positive” set to PCR-positive and culture-positive results; ^2^ (True-Pos + True-Neg)/Sum; ^3^ True-Neg/(True-Neg + False-Pos); ^4^ True-Pos/(True-Pos + False-Neg); ^5^ True-Pos/(True-Pos + False-Pos); ^6^ True-Neg/(True-Neg + False-Neg).

Building water systems that have been successfully disinfected often yield samples with results that are PCR-positive but spread plate culture-negative. Such results provide valuable and scientifically defensible validation evidence of effective hazard control under operating conditions. However, this result could also indicate presence of VBNC *Legionella* indicating that the treatment may have been not entirely effective.

## 5. Conclusions

Disease and injury caused by hazardous conditions associated with building water systems is serious and preventable. The way water is processed and used in buildings results in water quality degradation; therefore process management is necessary to prevent harm. Many successful applications of building water system management have been published. A consensus has developed regarding the most practical and effective process management system to prevent disease and injury from building water systems. Comparison of recently published directives, guidance and proposed standards indicate consistency in principle and implementation. The framework for process management of building water systems is firmly rooted in the hazard analysis and critical control point (HACCP) system, which is an adaptation of the extensively applied, and widely successful failure mode and effects (FMEA) system for process management.
